# Acute Bronchitis

**Published:** 1900-01

**Authors:** 


### Acute Bronchitis.

Potter recommends the following:

| | |
|---|---|
| Antimonii et potass. tart. | gr. 2. |
| Liquor ammon. acet. | oz. 4. |
| Spr. etheris nitrosi. | oz. 1. |
| Tinct. aconiti | dr. ½. |
| Syr. simplicis | q. s. oz. 6. |

M. Sig.: A teaspoonful every two or three hours in the first stage.

